# Cross-reactive human B cell and T cell epitopes between influenza A and B viruses

**DOI:** 10.1186/1743-422X-10-244

**Published:** 2013-07-26

**Authors:** Masanori Terajima, Jenny Aurielle B Babon, Mary Dawn T Co, Francis A Ennis

**Affiliations:** 1 Division of Infectious Diseases and Immunology, Department of Medicine, University of Massachusetts Medical School, 55 Lake Avenue North, Worcester, MA 01655, USA; 2 Division of Diabetes, Department of Medicine, University of Massachusetts Medical School, Worcester, MA, USA

**Keywords:** Influenza A virus, Influenza B virus, Cross-reactive B cell epitopes, Cross-reactive T cell epitopes, Hemagglutinin, Fusion peptide, Neuraminidase, Polymerase basic 1

## Abstract

Influenza A and B viruses form different genera, which were originally distinguished by antigenic differences in their nucleoproteins and matrix 1 proteins. Cross-protection between these two genera has not been observed in animal experiments, which is consistent with the low homology in viral proteins common to both viruses except for one of three polymerase proteins, polymerase basic 1 (PB1). Recently, however, antibody and CD4^+^ T cell epitopes conserved between the two genera were identified in humans. A protective antibody epitope was located in the stalk region of the surface glycoprotein, hemagglutinin, and a CD4^+^ T cell epitope was located in the fusion peptide of the hemagglutinin. The fusion peptide was also found to contain antibody epitopes in humans and animals. A short stretch of well-conserved peptide was also identified in the other surface glycoprotein, neuraminidase, and antibodies binding to this peptide were generated by peptide immunization in rabbits. Although PB1, the only protein which has relatively high overall sequence homology between influenza A and B viruses, is not considered an immunodominant protein in the T cell responses to influenza A virus infection, amino acid sequence comparisons show that a considerable number of previously identified T cell epitopes in the PB1 of influenza A viruses are conserved in the PB1 of influenza B viruses. These data indicate that B and T cell cross-reactivity exists between influenza A and B viruses, which may have modulatory effects on the disease process and recovery. Although the antibody titers and the specific T cell frequencies induced by natural infection or standard vaccination may not be high enough to provide cross protection in humans, it might be possible to develop immunization strategies to induce these cross-reactive responses more efficiently.

## Introduction

Human influenza is a highly contagious acute respiratory illness that is responsible for significant morbidity and excess mortality especially in the elderly and the very young worldwide. On average 5% to 20% of the population in the United States acquires influenza every year with more than 200,000 people hospitalized from influenza complications, while influenza-related deaths range from 3000 to 49,000 (“Seasonal Influenza: The Disease” by Centers for Disease Control and Prevention (http://www.cdc.gov/flu/about/disease/index.htm)). Causative agents, influenza A and B viruses, belong to the family *Orthomyxoviridae*[1]. Influenza C viruses cause milder respiratory illnesses than influenza A and B viruses [2]. They were originally distinguished by antigenic differences in their nucleoprotein (NP) and matrix 1 protein (M1). The type A influenza viruses are further divided into subtypes based on the antigenicity of surface glycoproteins, hemagglutinin (HA) and neuraminidase (NA). Currently there are 17 HA subtypes (H1-H17) and 10 NA subtypes (N1-N10) [3,4]. Influenza A viruses cause pandemic and seasonal epidemics, and influenza B viruses are responsible for widespread epidemics every 3 to 4 years [5]. Mortality rates are higher in epidemics caused by influenza A virus H3N2 subtypes than those by H1N1 subtypes or by influenza B viruses in recent years. Influenza A viruses have a broad host range including mammals and birds, while influenza B and C viruses are primarily pathogens of humans [5].

Influenza A and B viruses are more similar to each other than to influenza C viruses in terms of genome organization and protein homology [6-11]. Influenza A and B viruses have eight negative sense RNA segments as a genome, while influenza C viruses have seven [12]. These eight segments encode for more than eleven proteins with ten of them common between influenza A and B viruses [12,13]. The structure of influenza A and B virus particles are very similar by electron microscopy [14]. However, cross-protection between influenza A and B viruses was not observed in mouse experiments performed almost a half century ago [15]. Schulman and Kilbourne compared mice previously infected with influenza A virus H1N1 subtype or influenza B virus in an aerosol challenge experiment with influenza A virus H2N2 subtype and found no difference in pulmonary virus titers from one to four days after challenge (in a separate experiment, the same challenge dose killed 100% of previously uninfected mice and 13.3% of A/H1N1 subtype-immune mice, which suggested the presence of heterosubtypic immunity between A/H1N1 and A/H2N2 subtypes). They also found no difference in pulmonary virus titers after influenza B virus challenge between A/H1N1 subtype-immune mice and previously uninfected mice [15]. In recent papers influenza B virus infection was used as a negative control in experiments analyzing heterosubtypic immunity among influenza A virus subtypes in mice [16] and ferrets [17]. The challenge doses chosen in the mouse experiments were the lowest dose to kill 100% of unimmunized mice. It was stated in the text that influenza B virus immunization did not protect mice from influenza A virus challenge in pilot experiments, although in some experiments using wild type mice, influenza B virus-immune mice showed survival from 25% to 44% against A/H1N1 or A/H3N2 virus challenge (in the same experiments A/H1N1- or A/H3N2-immune mice showed survival rates from 86% to 100% against heterosubtypic challenge) [16]. In ferret experiments, infection with various influenza virus A/H1N1 strains or an influenza virus B strain did not produce cross-reactive antibody responses between influenza A and B viruses in hemagglutination inhibition or neutralization assays [17]. Overall the animal experiments are limited because of the absence of T cell studies, but the antibody studies and challenge results are consistent with the low amino acid homology between influenza A and B viral proteins [13]. One difference between infection in humans and animal experiments is that humans are infected and vaccinated with influenza virus repeatedly throughout their life while animals in these experiments experienced infection or vaccination only once or twice.

Table 1 compares the ten viral proteins common in influenza A and B viruses. Viral strains for comparison were selected from recent vaccine strains for which all viral protein sequences were available from GenBank. The NP and the M1 being used for distinguishing A and B types have 36% and 27% homology respectively. As expected from the presence of many subtypes in influenza A viruses, the HA and the NA have lower homology (18% for the HA and 20% for the NA) than the NP and the M1. Nonstructural protein 1 (NS1) and NS2/nuclear export protein (NEP) are in a similar range as the HA and the NA. There is almost no homology at the amino acid level between influenza A viruses’ matrix 2 protein (M2) and influenza B viruses’ BM2. Among three polymerases the polymerase basic 2 (PB2) and the polymerase acid (PA) have a similar homology range as NP, but the polymerase basic 1 (PB1) has a much higher homology, 58%, than any of the other influenza viral proteins [6,18].

**Table 1:** Amino acid homology between influenza A and B viral proteins

**Protein name**	**Size in influenza A**^**a**^	**Size in influenza B**^**b**^	**Amino acid (aa) identity**^**c**^
Polymerase basic 2 (PB2)	759 aa	770 aa	37%
Polymerase basic 1 (PB1)	757 aa	752 aa	58%
Polymerase acid (PA)	716 aa	726 aa	35%
Hemagglutinin (HA)	566 aa	584 aa, 585 aa^d^	18%
Nucleoprotein (NP)	498 aa	560 aa	36%
Neuraminidase (NA)	469 aa	466 aa	20%
Matrix protein 1 (M1)	252 aa	248 aa	27%
Matrix protein 2 (M2)^e^	97 aa	109 aa	7%
Nonstructural protein 1 (NS1)	219 aa, 230 aa^f^	281 aa, 282 aa^g^	14%
NS2/nuclear export protein (NEP)	121 aa	122 aa, 123 aa^h^	21%

^a^Amino acid sequences are from influenza A/California/07/2009(H1N1) (GenBank protein accession number: AFM72841, AFM72840, AFM72839, AFM72832, AFM72836, AFM72835, AFM72833, AFM72834, AFM72837 and AFM72838), A/Brisbane/10/2007(H3N2) (ACI26328, ACI26326, ACI26325, ACI26318, ACI26322, ACI26321, ACI26319, ACI26320, ACI26323 and ACI26324).

^b^Amino acid sequences are from influenza B/Wisconsin/01/2010 (B/Yamagata/16/88-lineage) (AFH57963, AFH57962, AFH57961, AFH57953, AFH57958, AFH57957, AFH57954, AFH57955, AFH57959 and AFH57960) and B/Brisbane/60/2008 (B/Victoria/2/87-like lineage) (AFH57919, AFH57918, AFH57917, AFH57909, AFH57914, AFH57913, AFH57910, AFH57911, AFH57915 and AFH57916).

^c^Conserved amino acids among four viruses. Denominators are the proteins of A/California/07/2009(H1N1).

^d^584 aa for B/Wisconsin/01/2010 and 585 aa for B/Brisbane/60/2008.

^e^Corresponding influenza B virus protein is BM2 [19].

^f^219 aa for A/California/07/2009(H1N1) and 230 aa for A/Brisbane/10/2007(H3N2).

^g^281 aa for B/Wisconsin/01/2010 and 282 aa for B/Brisbane/60/2008.

^h^122 aa for B/Wisconsin/01/2010 and 123 aa for B/Brisbane/60/2008.

In this review, we will summarize recent reports including ours identifying B and T cell epitopes cross-reactive between influenza A and B viruses and re-evaluate existing data on T cell responses to influenza A viruses in view of cross-reactivity to influenza B viruses. We will refer to other review articles dealing with B and T cell epitopes shared within influenza A viruses [20-25] and concentrate on cross-reactivity between influenza A and B viruses in this report.

## Review

### B and T cell epitopes in the fusion peptide of the HA

**Figure 1:**
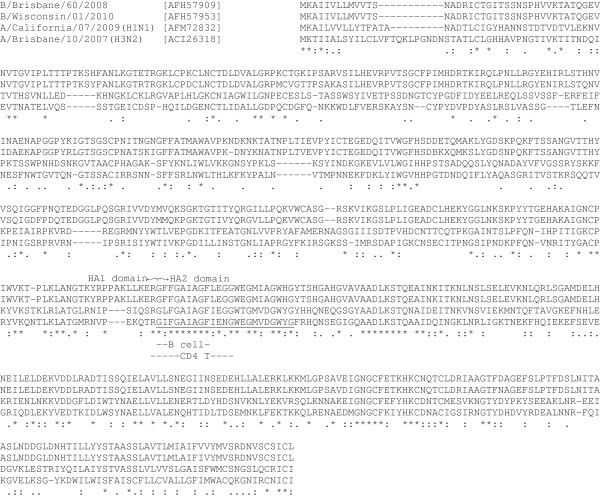
**Comparison of the HA protein amino acid sequences among influenza A and B viruses. **Multiple alignment was performed by CLUSTALW (available at http://www.genome.jp/tools/clustalw/). Protein accession numbers are shown in brackets next to influenza virus strain names. The fusion peptide is underlined [28]. B cell and CD4^+ ^T cell epitopes are also shown.

Although the overall homology between the HAs of influenza A and B viruses is only 18% (Table 1), there is a short stretch of well-conserved peptide (Figure 1) [26-28], which overlaps with the fusion peptide (underlined in Figure 1) [5,28]. Immunogenicity of the fusion peptide was first analyzed in 1982 by Atassi and Webster [29]. They found that sera obtained from infected humans, and from rabbits and goats immunized with adjuvanted HA protein had binding antibodies cross-reactive to the fusion peptides. Immunization with the synthetic peptides (GLFGAIAGFIE for A virus and GFFGAIAGFIE for B virus) induced peptide specific antibodies in rabbits, but these antibodies (antisera) were not neutralizing. Peptide immunization (with complete Freund’s adjuvant) did not protect mice from infection with either influenza A or B viruses (ten 50% lethal dose (LD_50_)), although monoclonal antibodies established from immunized mice were neutralizing in vitro (the antisera were not) [29].

More than 20 years later there is renewed interest in the fusion peptide as a target of antibodies [27,30-32]. Chun et al. analyzed the NCBI influenza virus resource database and identified 14-amino acid peptides in the fusion peptide, G(L/I/F)FGAIAGFIE(G/N)GW, which have only two positions with amino acid variations among all influenza A and B viruses [27]. Antibodies (called Uni-1 antiserum) raised in rabbits against one of these peptides (conjugated to the keyhole limpet hemocyanin (KLH) mixed with Freund’s adjuvant), GLFGAIAGFIEGGW, were able to bind to both influenza A (subtypes H1-H13 were tested) and B viruses [27]. Contrary to the previous report by Atassi and Webster, the Uni-1 antiserum showed neutralizing activity against live influenza A/H1N1 and A/H3N2 viruses and an A/H5N1 pseudotype virus in vitro through inhibition of viral fusion with the cells [31] (the Uni-1 antiserum was not tested against influenza B virus).

Stanekova et al. showed that peptide immunization with the fusion peptide (the first 38 amino acids of HA2 domain of influenza A/Mississippi/1/85(H3N2) strain conjugated to the KLH mixed with Freund’s adjuvant) produced anti-fusion peptide antibodies and protected mice against mild challenge with homologous and heterologous (H1N1 or H7N1) viruses (1 and 2 LD_50_) [32,33]. Prabhu et al. established a monoclonal antibody from mice immunized with recombinant H5 HA protein (an uncleaved HA0 mixed with adjuvant) whose epitope was mapped to the fusion peptide (GLFGAIAGF). This antibody, MAb 1C9, had no detectable hemagglutination inhibition or neutralization titer, but inhibited cell fusion in vitro and protected mice against live virus challenge with H5N1 subtype (5 LD_50_) [30]. Liu et al. reported that DNA vaccination with full-length HA cDNA (seasonal and 2009 pandemic H1) did not induce antibodies binding to GLFGAIAGFIE peptide in mice [34].

In these four mouse experiments BALB/c mice were used for immunization, suggesting that anti-fusion peptide antibody production may depend on immunization strategy. Immunization with purified whole HA protein induced a neutralizing anti-fusion peptide antibody [30]. Immunization with the fourteen-amino acid peptide [29] and the thirty eight-amino acid peptide [32,33] also induced anti-fusion peptide antibody. The former failed to protect mice against challenge, while the latter protected, which may be explained by the difference of challenge doses (10 versus 1 and 2 LD_50_). Hybridomas from the former immunization did produce the fusion-peptide specific antibodies which were neutralizing in vitro [29]. In contrast DNA immunization failed to induce detectable antibody response specific to the fusion peptide [34].

Liu et al. also reported that they did not detect antibodies binding to GLFGAIAGFIE peptide in the sera of adult humans, which is different from the report by Atassi and Webster [29], although subjects were very different temporally (1982 versus 2008–9 before 2009 H1N1 pandemic) and geographically (US versus China).

We identified a CD4^+^ T cell epitope which was also located in the fusion peptide when we were analyzing T cell responses to the H2 HA [35]. We detected interferon-γ responses to the H2 HA peptides in PBMCs obtained from donors who were born years after influenza A virus H2N2 subtype disappeared from circulation in the human population (it was replaced by H3N2 subtype in 1968) suggesting the presence of highly conserved T cell epitopes in the HA of influenza A viruses. Further analyses identified a CD4^+^ T cell epitope (RG(L/I/F)FGAIAGF(I/L)E(G/N)G)^a^ in the fusion peptide. Since the fusion peptide is highly conserved in both influenza A and B viruses, we decided to test the cross-reactivity of a CD4^+^ T cell line generated from one of the donors’ PBMCs. As expected the T cell line secreted interferon-γ and tumor necrosis factor-α after stimulation with influenza A/H1N1 (seasonal and 2009 pandemic), A/H2N1, A/H3N2, A/H5N1 and B viruses and lysed target cells infected with these viruses or pulsed with the purified HA proteins. The epitope is likely to be restricted by the HLA-DR (the epitope peptide is likely to be restricted by the HLA-DRB1*09 allele but can also bind to the HLA-DRB1*01 allele) [35]. CD4^+^ T cells can control influenza virus infection by producing inflammatory cytokines and chemokines, directly lysing infected cells, and by helping B cells and CD8^+^ T cells [36,37]. Wilkinson et al. showed that the frequencies of preexisting influenza-specific CD4 T cells correlated with disease protection against experimental challenge when the subjects did not have hemagglutination inhibition antibodies to the challenge strain [38].

A CD8^+^ T cell epitope was also identified in the fusion peptide. Gianfrani et al. identified peptides which had an HLA-A2.1 binding motif and bound to the HLA-A2.1 molecule (GLFGAIAGFI) within the fusion peptide based on the sequence of influenza virus A/Puerto Rico/8/34(H1N1) strain [39]. This HLA-A2.1-binding peptide, however, was not considered an epitope in their report because immunization with the peptide did not induce cytotoxic T cells response in HLA-A2.1/Kb transgenic mice. Several years later Kosor Krnic et al. generated a peptide/MHC class I tetramer with this peptide and detected the peptide-specific CD8^+^ T cells in humans proving that the peptide is indeed the HLA-A2.1-restricted CD8^+^ T cell epitope [40]. Although neither of these papers tested the cross-reactivity of this CD8^+^ T cell epitope, it may not be cross-reactive to influenza B viruses when we consider the amino acid change from leucine/isoleucine to phenylalanine at one of the anchor residues (2nd position).

### B cell epitope in the stalk region of the HA

A protective antibody epitope cross-reactive to influenza A and B viruses was also located in the stalk region of the HA. Dreyfus et al. cloned human monoclonal antibodies from B cells from volunteers vaccinated with the seasonal influenza vaccine and one of these antibodies, CR9114, bound to both influenza A and B viruses tested. Although the monoclonal antibody neutralized only influenza A viruses in vitro, it protected mice against both influenza A and B viruses challenge. This monoclonal antibody was found to bind to the stalk (stem) region of the HA [41].

The stalk-specific antibodies tend to have broader neutralizing activity than the conventional globular head-specific antibodies among influenza A viruses and are being studied extensively (reviewed in [21]) including immunization strategies to efficiently induce the stalk-specific antibodies [42,43]. As mentioned in the introduction, we will not discuss details about influenza virus A subtype-cross-reactive antibodies here, but it should be pointed out that these antibodies bind to conformational epitopes [44] and that influenza A/New Jersey/1976(H1N1) and A/California/04/2009(H1N1) vaccines (inactivated vaccines), both of which are of swine-origin, boosted the stalk-specific antibodies in humans suggesting that vaccines containing viruses whose globular head of the HA are substantially different from seasonal strains are capable of boosting titers of the stalk-specific antibodies [45]. Prime/boost vaccination with plasmid DNA encoding the HA cDNA followed by seasonal vaccine also induced the stalk-specific antibodies in a number of different animals including non-human primates [46]. HA protein without the globular head (containing the fusion peptide) is also being studied as an vaccine antigen to induce the stalk-specific antibodies [42,47,48]. One study estimated that ~0.001% of the total immunoglobulin G were these stalk-specific antibodies (subtype-cross-reactive among Group 1 HAs) [49]. How common are antibodies like CR9114 among the stalk-specific antibodies is not known.

It is interesting and important to know if these immunization strategies can also induce the fusion peptide-specific antibodies.

### B cell epitope in the NA

**Figure 2:**
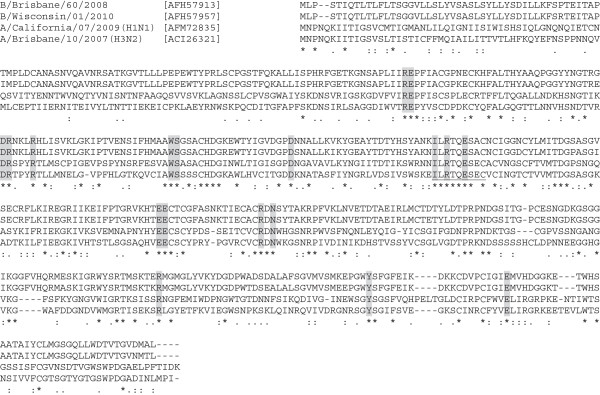
**Comparison of the NA protein amino acid sequences among influenza A and B viruses. **Multiple alignment was performed by CLUSTALW (available at http://www.genome.jp/tools/clustalw/). Protein accession numbers are shown in brackets next to influenza virus strain names. The conserved peptide studied by Gravel et al. [51] is underlined, and amino acid residues considered to be involved in the enzymatic activity [52] are shaded.

Some antibodies against the NA can bind near its catalytic site and inhibit virus release from infected cells and these epitopes are conformational [50]. A short stretch of well-conserved peptide among influenza A and B viruses was identified in the NA by Gravel et al. [51], which includes residues necessary for the enzymatic function [52]. The peptide shows amino acid sequence variation only at one position [51] (Figure 2). Immunization with a peptide designed based on the type A virus sequence (the nine-amino acid long conserved peptide was conjugated to the KLH with a spacer) induced antibodies cross-reactive to the NA proteins from both influenza A and B viruses in rabbits [51]. Functionality of these antibodies against influenza viruses has not been reported. Studies showed that natural infection induced anti-NA antibodies in humans [53] and that vaccination with trivalent inactivated influenza vaccine (TIV) or live attenuated vaccine induced anti-NA antibodies [50]. It is not known if antibodies specific to this NA peptide are induced in humans by natural infection or vaccination.

### Conservation of T cell epitopes in PB1 among influenza A and B viruses

Among influenza A and B viral proteins only polymerase basic 1 has relatively high overall sequence homology compared to other viral proteins (Table 1) [6,18]. PB1 was not considered to be an immunodominant protein in T cell responses against influenza viruses; however, a recent study reported that PB1 had more CD4^+^ and CD8^+^ T cell epitopes than any other influenza A virus proteins in humans [54]. Of 25 CD8^+^ T cell epitopes restricted by HLA-A1, -A2, -A3, -A24, -B7 and -B44 supertypes which are highly conserved among H1N1 (seasonal and 2009 pandemic), H2N2, H3N2, H5N1, H7N7 and H9N2 subtypes, six epitopes are on PB1. Only nucleoprotein has more epitopes (eight) [55].

In mice, a DNA vaccine expressing the PB1 protein from A/Puerto Rico/8/34(H1N1) strain protected against homologous challenge (although the challenge was relatively modest with 1 LD_50_) [56]. Anti-PB1 antibodies were produced in the vaccinated mice. Since there is an example of a neutralizing antibody which can act within cells in Listeria monocytogenes infection [57], it is possible that these anti-PB1 antibodies mediate protection by affecting influenza virus-infected cells, and not by neutralizing cell-free virions. But, it is also reasonable to consider that T cell responses against PB1 (both CD4^+^ and CD8^+^) may have been induced in vaccinated mice and contributed to the observed protection.

We looked at amino acid sequence conservation in T cell epitopes in the PB1 reported in the literature (all reported T cell epitopes in the influenza virus PB1 were identified using influenza A viruses or their amino acid sequences). The result is summarized in Table 2. As expected from the 58% amino acid identity, 14 of the 32 well-characterized T cell epitopes (shown in the top half of the table) were identical or only had conservative amino acid variations. Twelve were CD8^+^ T cell epitopes and two were CD4^+^ T cell epitopes. Most of the CD8^+^ T cell epitopes are restricted by HLA-A1, -A2, -A3 or -A24 supertypes [58] and the two CD4^+^ T cell epitopes are restricted by HLA-DR supertype [59] suggesting that these CD8^+^ and CD4^+^ T cell epitopes are expected to be recognized by a majority of human population. Eleven of the 14 conserved T cell epitopes were clustered in two regions, amino acid 404–422 and 471–517. The same regions also contain several less-characterized T cell epitopes (the bottom half of the table). The presence of these epitopes suggests that there are cross-reactive T cell responses between the PB1 proteins of influenza A and B viruses.

**Table 2:** Conservation of T cell epitopes identified in the PB1 of influenza A and B viruses

**Peptide location**^**a**^	**Peptide sequence of influenza A PB1**^**b**^	**Corresponding peptide in influenza B PB1**^**c**^	**Amino acid identity**^**d**^	**Restricting allele**	**Reference**
1-15	MDVNPTLLFLKVPAQ	*NI**YF**ID**(I/V)*	8/15	DR supertype	[54]
7-14	LLFLKVPA	F**ID**(I/V)	4/8	A2 supertype	[54]
**30-38**	**YSHGTGTGY**	*********	**9/9**	**A1 supertype**	[55]
**41-49**	**DTVNRTHQY**	***I*****	**8/9**	**A26**	[60]
92-106	MAFLEESHPGIFENS	LDRMD*E***L*QAA	5/15	DR supertype	[54]
166-174	FLKDVMESM	*CQ*IID*L	3/9	A2	[60]
238-246	RRAIATPGM	******A*I	7/9	B27	[61]
254-262	FVEALARSI	V**N**KN*	5/9	A2 supertype	[54]
257-265	TLARSICEK	N**KN***N	5/9	A3 supertype	[54]
**263-271**	**CEKLEQSGL**	**N******	**8/9**	**B44**	[54]
316-330	RMFLAMITYITRNQP	*I****TER***DS*	9/15	DR supertype	[54]
347-355	KMARLGKGY	*I******F	7/9	B62	[60]
349-357	ARLGKGYMF	******F*I	7/9	B27	[60]
357-364	FESKSMKL	IT**TKR*	3/8	B44	[54]
**404-418**	**SPGMMMGMFNMLSTV**	***************	**15/15**	**DR supertype**	[54]
**407-415**	**MMMGMFNML**	*********	**9/9**	**A2 supertype**	[54]
**408-422**	**MMGMFNMLSTVLGVS**	**************A	**14/15**	**DR supertype**	[54]
**412-421**	**FNMLSTVLGV**	**********	**10/10**	**A2 supertype**	[54]
**413-421**	**NMLSTVLGV**	**********	**9/9**	A*0201	[39]
**471-480**	**KLVGINMSKK**	**L*(*/V)*****	**8~9/10**	**A3 supertype**	[55]
**488-497**	**GTFEFTSFFY**	*M*****M**	**8/10**	**A3 supertype**	[55]
**489-497**	**TFEFTSFFY**	M*****M**	**7/9**	**A1 supertype**	[54]
490-497	FEFTSFFY	*****M**	7/8	B44	[54]
**496-505**	**FYRYGFVANF**	***D***S**	**8/10**	**A24 supertype**	[55]
501-509	FVANFSMEL	**S**A**(*/I)	6~7/9	A2 supertype	[54]
**505-514**	**FSMELPSFGV**	*A**(*/I)*****	**8~9/10**	**A2 supertype**	[54]
**509-517**	**LPSFGVSGI**	(*/I)*****A*V	**6~7/9**	**B7**	[54]
540-548	GPATAQMAL	******T*I	7/9	B7	[60]
566-574	TQIQTRRSF	SKVEGK*MK	1/9	B62	[60]
590-599	LVSDGGPNLY	**A*****I*	8/10	A1 supertype	[55]
591-599	VSDGGPNLY	*A*****I*	7/9	A1	[62]
741-749	AEIMKICST	EKA*AHLGE	1/9	B44	[54]
21-38	TFPYTGDPPYSHGTGTGY	******V***********	17/18	CD8	[63]
43-60	VNRTHQYSEKGKWTTNTE	*I***E**N***QY(I/V)SD(V/I)	9/18	?	[63]
57-73	TNTETGAPQLNPIDGPL	(I/V)SD(V/I)**CTM(V/I)D*TN***	6/17	?	[63]
64-82	PQLNPIDGPLPEDNEPSGY	(T/A/V)MVD*TN**********A*	12/19	?	[63]
86-103	DCVLEAMAFLEESHPGIF	******LDRMD*E***L*	11/18	?	[63]
123-140	TQGRQTYDWTLNRNQPAA	******F***VC******	15/18	?	[63]
270-287	GLPVGGNEKKAKLANVVR	*************S*A*A	15/18	?	[63]
316-333	RMFLAMITYITRNQPEWF	*I****TER***DS*I**	11/18	?	[63]
395-428	LLIDGTASLSPGMMMGMFNMLSTVLGVSILNLGQ	FNEE***********************AA*GIKN	24/34	?	[64]
**402-419**	**SLSPGMMMGMFNMLSTVL**	******************	**18/18**	**CD4**	[63]
410-426	GMFNMLSTVLGVSILNL	************AA*GI	13/17	?	[63]
417-433	TVLGVSILNLGQKRYTK	*****AA*GIKNIGNKE	6/17	CD4	[63]
432-449	TKTTYWWDGLQSSDDFAL	GNKE*L************	13/18	CD4	[63]
447-463	FALIVNAPNYAGIQAGV	***F***KDE*TCME*I	8/17	?	[65]
470-486	CKLVGINMSKKKSYINR	***L*(*/V)********C*E	13~14/17	?	[63]
498-514	RYGFVANFSMELPSFGV	*D***S**A**(*/I)*****	13~14/17	CD8	[63]
505-521	FSMELPSFGVSGINESA	*A**(*/I)*****A*V****	13~14/17	?	[63]
548–564	LQLFIKDYRYTYRCHRG	I****A******K****	14/17	?	[65]
562-579	HRGDTQIQTRRSFELKKL	****SKVEGK*MKII*E*	7/18	?	[63]
705-722	YRRPVGISSMVEAMVSRA	**K***QH**L***AH*L	11/18	CD8	[63]

^a^Based on the PB1 of A/Puerto Rico/8/34(H1N1) (NP_040985).

^b^Amino acid sequence in the original publication.

^c^B/Wisconsin/01/2010 (B/Yamagata/16/88-lineage) and B/Brisbane/60/2008 (B/Victoria/2/87-like lineage).

^d^Embolden when all amino acids are identical or when the observed amino acid variation in B viruses does not decrease the SYFPEITHI binding score (http://www.syfpeithi.de/bin/MHCServer.dll/EpitopePrediction.htm).

Commercial TIVs do not contain detectable levels of PB1 by two-dimensional high-performance liquid chromatography [66,67], and in mice TIV did not induce PB1-specific CD4^+^ T cell responses [68]. It is not known if live attenuated vaccines induce PB1-specific CD4^+^ and CD8^+^ T cell responses.

Since CD4^+^ and CD8^+^ T cell responses tend to be more subtype-cross-reactive than antibody responses [24,25,69], there are a variety of vaccine formulations designed to induce CD4^+^ and CD8^+^ T cell responses against influenza viral proteins (reviewed in [25]). There are two DNA vaccine studies which used the PB1 gene or gene segments encoding peptide epitopes as a vaccine antigen. One is a plasmid DNA vaccine with full-length PB1 cDNA described above [56], and the other is a plasmid DNA vaccine made of 20 epitopes restricted by many different HLA-DR alleles designed to induce CD4^+^ T cell responses in diverse ethnic groups [70]. In the latter study focusing on CD4^+^, not CD8^+^, T cell responses, DNA vaccination alone failed to induce detectable levels of epitope-specific CD4^+^ T cell responses in HLA-DR4 transgenic mice, however, the vaccinated mice showed protection against a challenge by A/Puerto Rico/8/34(H1N1) (4 LD_50_), although the relative contribution of CD4^+^ T cell responses to the PB1 epitopes in protection was not known [70].

## Conclusions

The data we reviewed here show that B and T cell cross-reactivity exists between influenza A and B viruses, which may have modulatory effects on the disease process and recovery. Although the antibody titers and the specific T cell frequencies induced by natural infection or standard vaccination may not be high enough to provide cross protection, it might be possible to develop immunization strategies to induce these cross-reactive responses more efficiently. Specifically, immunization strategies to induce the stalk-specific antibodies might also be efficacious for induction of the fusion peptide-specific antibodies (in addition to inducing stalk-specific antibodies, some of which may be cross-reactive to both influenza A and B viruses). DNA vaccine or recombinant viral vectors expressing PB1 cDNA alone may not be protective against high dose challenge, but may enhance protection against influenza conferred by immune responses to HA or NA.

## Endnote

^a^The conserved sequence reported in the paper [35] contained errors. Authors’ Correction will be published in the Journal of Virology, volume 87, issue 16 in 2013.

## Abbreviations

PB1: Polymerase basic 1; NP: Nucleoprotein; M1: Matrix 1 protein; HA: Hemagglutinin; NA: Neuraminidase; NS1: Nonstructural protein 1; NEP: Nuclear export protein; M2: Matrix 2 protein; PB2: Polymerase basic 2; PA: Polymerase acid; LD50: 50% lethal dose; KLH: Keyhole limpet hemocyanin.

## Competing interests

The authors declare that they have no competing interests.

## Authors' contributions

MT wrote the initial draft. All authors discussed, read and approved the final manuscript.
